# Associations among Orthodontic History, Psychological Status, and Temporomandibular-Related Quality of Life: A Cross-Sectional Study

**DOI:** 10.1155/2022/3840882

**Published:** 2022-05-28

**Authors:** Jia-Qi Liu, Yi-Dan Wan, Tian Xie, Tao Miao, Jun Wang, Xin Xiong

**Affiliations:** ^1^National Clinical Research Center for Oral Diseases, State Key Laboratory of Oral Diseases, Department of Orthodontics, West China Hospital of Stomatology, Sichuan University, Chengdu, Sichuan, China; ^2^Department of Nursing, West China Hospital of Stomatology, Sichuan University, Chengdu, Sichuan, China; ^3^Department of Stomatology, The Shenzhen Second People's Hospital, The First Affiliated Hospital of Shenzhen University Health Science Center, Shenzhen, China

## Abstract

**Objectives:**

This cross-sectional study aimed to evaluate the associations among orthodontic history, psychological status, and temporomandibular-related quality of life.

**Methods:**

A questionnaire was developed and distributed to students in a local college, containing questions about demographic information, the Patient Health Questionnaire-4 (PHQ-4), the Fonseca anamnestic index, and the Oral Health Impact Profile for Temporomandibular Disorders (OHIP-TMD). The respondents were divided into with orthodontic history (OS) group and without OS group. Binary logistic regression and multiple linear regression were performed for statistical analysis.

**Results:**

A total of 531 valid questionnaires were collected, covering 161 participants with OS and 370 participants without OS. No statistically significant differences were observed in the scores of PHQ-4 between the two groups. There was statistical difference in the prevalence of TMD (with OS group, 54.66%; without OS group, 40.81%) and the mean value ( ± standard deviations) of the scores of OHIP-TMD (with OS group, 9.64 ± 12.36; without OS group, 6.64 ± 10.79) (*p* < 0.05). After adjusting confounding factors, participants with OS have worse temporomandibular-related quality of life and a higher risk of having TMD than the participants without OS.

**Conclusions:**

Orthodontic history was related with the higher prevalence of TMD and worse temporomandibular-related quality of life, but not related with psychological distress, and the cause-and-effect relationship needs further exploration.

## 1. Introduction

Temporomandibular disorder (TMD) is a common disease that often affects the temporomandibular joint (TMJ), the masticatory muscles, and associated structures [1]. TMD is one of the musculoskeletal conditions that most commonly result in discomfort and pain [2]. It gives rise to significant personal burdens, whether physical or emotional, impeding the patient's quality of life [3]. Meanwhile, TMD is a highly prevalent condition, affecting 13.10%∼75.78% of the general population [4, 5], which females are more likely to suffer from [6].

In recent years, the number of people seeking orthodontic treatment shows an increasing trend. Orthodontic treatment could align the teeth, improve the patients' smile, and might improve the patients' oral health-related quality of life [7]. However, the effect of orthodontic treatment on the TMJ remains controversial in the literature [8].

The TMJ, as a component of the masticatory system, could be affected by the occlusal changes caused by the orthodontic treatment [9]. After proper orthodontic treatment, condition in the TMJ area of some patients with malocclusions might be improved. However, improper treatment plans and procedures might provoke or aggravate patients' temporomandibular symptoms [7].

Patient-reported outcomes are recommended in the assessment and management of patients with TMD [10]. The Oral Health Impact Profile for TMD (OHIP-TMD) questionnaire, which is an oral health-related quality of life (OHRQoL) instrument, is widely used to measure the impact of TMDs on the patients' well-being from multiple aspects [11]. Compared with healthy people, TMD patients showed both physically and psychosocially poorer temporomandibular-related quality of life [12]. Compared with nonpainful TMDs, painful TMD patients showed poorer OHRQoL [13]. The risk factors for poorer temporomandibular-related quality of life are still needed to be investigated.

Many studies have been conducted on the relationships between orthodontic treatment and TMD [14, 15], but few previous studies have investigated the effect of orthodontic history (OS) on the patients' temporomandibular-related quality of life and psychological status. Therefore, this study's objectives were to evaluate the associations among orthodontic history, psychological status, and temporomandibular-related quality of life. The null hypothesis is that OS is not associated with psychological distress or worse temporomandibular quality of life.

## 2. Materials and Methods

The protocol of this research was approved by the Institutional Review Board of West China Hospital of Stomatology.

### 2.1. Participants

The sample size was calculated using the G*∗*power software (version 3.1.9, Germany), assuming *α* (two‐tailed) = 0.05, level of *β* = 0.05 (95% power), effect size = 0.5, and allocation ratio = 3 : 1 (without OS to with OS), which was determined based on the results of our pilot study. The minimal sample size required was 280 participants.

A total of 637 students from a local university were asked to fill the survey questionnaires. The enrollment was via convenience sampling at public facilities in campus such as the classrooms, libraries, and student canteens. All participants were consent for the use of the data recorded. The exclusion criteria were as follows: (1) participants were currently under orthodontic treatment; (2) participants had a history of orthognathic treatment; and (3) participants had a history of systematic diseases or psychiatric illness. Participants who fulfilled one of the exclusion criteria were not asked to fill the questionnaire. Besides, the relevant questions regarding the exclusion criteria were developed in the questionnaire for reassurance.

### 2.2. Data Collection

The questionnaire consists of four parts. The first part is about the exclusion criteria and the demographic characteristics, including sex, age, education background, family per capita monthly income, major/occupation, and OS.

The second part is the Patient Health Questionnaire-4 (PHQ-4), a combination of the Patient Health Questionnaire-2 (PHQ-2) and the Generalized Anxiety Disorder-2 scale (GAD-2). PHQ-2 could be used for screening depression and GAD-2 for anxiety [16]. The questionnaire consists of four items and was designed to be used in general practice with accurate results [16, 17]. Item scores range from 0 (not at all) to 3 (nearly every day). The total possible score of PHQ-4 ranges from 0 to 12; higher scores indicate greater severity. A score of more than 2 on PHQ-2 and GAD-2 subscale indicates the presence of depression and anxiety [18].

The third part is the Fonseca anamnestic index (FAI) questionnaire, a 10‐item multidimensional instrument that assesses pain frequency, psychological distress, jaw function limitations, and parafunctional behaviors associated with TMD [19]. It was developed based on the Helkimo index and has been mooted as a simple, low‐cost, and patient‐reported TMD assessment tool to diagnose TMD [20]. The Chinese version had accepted reliability and good validity [21]. Subjects were required to score the individual items on a 3‐point response scale with no, sometimes, and yes conferring 0, 5, and 10 points, respectively. Summary scores for all 10 items were subsequently computed and used to classify the severity of TMD. A total score no more than 15 was considered TMD-free and more than 15 was considered as having TMD.

The last part is the OHIP-TMD scale. It consists of 22 items (two items were newly added in the OHIP-TMD: Have you had difficulties in opening and closing your mouth? and Have you felt speech was painful because of problems with your teeth, mouth, dentures, or jaws?) grouped into seven domains to describe the functional limitation (items 1 and 2), physical pain (items 3–7), psychological discomfort (items 8–11), physical disability (items 12 and 13), psychological disability (items 14–18), social disability (items 19 and 20), and handicap (items 21 and 22). The response is a five-point Likert format: never, hardly ever, occasionally, fairly often, and very often (equivalent to scores of 0–4) [12].

### 2.3. Statistical Analysis

Continuous variables were presented as the mean ± standard deviation, and categorical variables were as frequency or percentage. The *t*-test or Mann–Whitney *U* test was performed for continuous variables. The Pearson chi-square test or Fisher's exact test was performed for categorical variables. Demographic characteristics were compared to determine whether there were statistical differences in the sample distribution. The presence of OS was considered as binary independent variable. Participants with OS were divided into With OS group, and those without OS were divided into Without OS group. Depression, anxiety, and TMD were considered as binary dependent variables, and the scores of TMD-OHIP were considered as continuous dependent variable. Binary logistic regression models were performed for binary variables, and multiple linear regression models were performed for continuous variable. Three models for each outcome variable were constructed: crude model with no adjusted covariates; model 1 with adjusted specific covariates, which were selected based on their associations with the outcomes of interest or a change in effect estimate of more than 10%; model 2 with all the covariates adjusted.

## 3. Results

A total of 531 questionnaires were collected, and the response rate was 83.36%. 161 participants (30.32%) had OS, while 370 (69.68%) did not (Table 1). There was no statistical difference in age between the two groups. The proportion of females in the With OS group was much higher than that in the Without OS group. The With OS group had higher income and greater proportion of medical students than the Without OS group (Table 1).

The PHQ-4 total scores were 3.17 (±2.28) in the With OS group and 2.90 (±2.69) in the Without OS group, and the data were not statistically significant (Table 2). Binary regression analysis showed there was no correlation between orthodontic history and the presence of depression or anxiety (Table 3).

There was no adjust I model since no covariate changed the estimates of OS on the presence of anxiety and depression by more than 10%.

There was a statistical difference in the prevalence of TMD diagnosed by the FAI between the two groups. The mean value (±SD) of the scores of FAI was 22.08 (±18.09) in the With OS group, which was higher than that of 17.70 (±16.91) in the Without OS group (Table 4). Binary regression analysis showed a higher presence of TMD in the group with OS (OR 1.75, 95% CI: 1.20–2.54). Sex caused a change in the effect estimate of more than 10%, and after the adjustment, the association between OS and prevalence of TMD was still significant (Table 5).

There was a statistical difference in the total scores of TMD-OHIP between the two groups (Table 4). For the physical pain, psychological discomfort, physical disability, psychological disability, and social disability domains, there were statistical differences between the two groups (all *p* < 0.05). Multiple linear regression analysis showed people with OS had higher TMD-OHIP scores (*β* 3.00, 95% CI: 0.91–5.09). Besides, income caused a change in effect estimate of more than 10%, and after the adjustment, the value of *β* turned higher than the nonadjusted model (Table 6).

## 4. Discussion

The study evaluated the associations among orthodontic history, psychological distress, and TMD in 531 participants. The results showed that there was an assured correlation between the orthodontic history and TMD.

In this study, there were more women in the With OS group, which is in line with the trend that females are more inclined to seek orthodontic treatment [22–24]. Orthodontic treatments are not usually covered by dental insurance, and the cost is a significant out-of-pocket expenditure for most families [23]. Therefore, it is reasonable that participants in the With OS group had higher income. A larger proportion of participants in the With OS group majored in medicine. Compared with nonmedical students, medical students have more oral health knowledge and might pay more attention to their oral health. Also, they were more likely to receive orthodontic treatment.

The mean score of PHQ-4 in this study was close to the previously reported value (2.98) among 934 college students in America [25]. One randomized controlled study conducted in adolescents reported that, in a short term, orthodontic treatment could improve the patients' self-concept, which is how someone perceives themselves [26]. However, many studies reported the treatment had no influence on the psychological well-being of the patients over the long term [27, 28]. Our study had similar results with most previous studies. The reason might be that psychological well-being could be affected by many factors such as genetic factors, hormones, and psychological trauma. One single common dental treatment might not play a vital role in the development or relief of mental distress.

Compared with similar research among Singaporean college students [29], the prevalence of TMD was similar (45.01% vs. 42.62%), but the total score of participants in our study (Supplementary Table 1) was relatively lower, indicating better temporomandibular quality of life. However, another research among Australian chiropractic students reported even better quality of life with the mean total score of 1.3 [30]. These differences may be attributed to the sample size, regional practice, or population differences.

The relationship between malocclusions and temporomandibular disorders was controversial in the literature [31]. Several observational studies reported that specific malocclusions, including posterior crossbite, anterior open bite, and lingual tipping deep overbite, were weakly associated with TMD [32, 33]. Based on the weak association rather than causation, the effect of orthodontic treatment on the temporomandibular was considered neutral in the literature [34].

Our results revealed that patients with orthodontic history had higher prevalence of TMD and worse temporomandibular-related quality of life. Since this was a cross-sectional study, no cause-and-effect relationship could be constructed. However, there might be several reasons for these interesting results. Yap et al. reported that two-thirds of subjects seeking for orthodontic treatment had TMD-related symptoms [35], which was relatively higher than the general population [29]. Considering the neutral effect of orthodontic treatment, the patients with orthodontic history might have a higher prevalence of TMD than the general population as well.

Another reason might be the patients' self-awareness of occlusion and temporomandibular condition. Orthodontic treatment is a long process usually lasting for one to two years. During the treatment, orthodontists might give the patients education about ideal occlusion and temporomandibular health [36]. Therefore, patients might be more accessible to knowledge about occlusion and temporomandibular health and be likely to rate low about their own occlusion and temporomandibular health. To validate or contradict this assumption, studies could be conducted to compare the difference of knowledge about TMJ and occlusion in patients before, under, or after orthodontic treatment. Last but not least, the negative effect of improper orthodontic treatment on the TMJ cannot be neglected [37]. Because of the nature of the study design, participants included in this study might receive orthodontic treatment of different levels from dentists with different education background [38], which was close to the real-world situation. There might be a chance that some participants might receive improper treatment and get their temporomandibular health compromised. This is only an assumption, and further studies could be conducted to contradict.

Through binary regression analysis, female was found to associate with higher prevalence of TMD, in accordance with previous studies [39, 40]. This might be the result that TMJ is a potential target organ for estrogen, which promotes arthropathy and fascial pain [41]. Besides, adjustment of income made the estimated effect of OS on temporomandibular quality of life greater than the nonadjusted model. People with higher income, which was more common in the With OS group, are more easily accessible to dental health care [42]. Higher income plays a promoting role in oral health; therefore, it was reasonable that the estimated effect turned greater after adjusting the income inequality.

A major strength of this study was the relatively large sample size, which might improve the reliability of the results. However, there were several limitations as well. First of all, this was a cross-sectional study and no causal relationship could be established. By asking the With OS participants about their temporomandibular-related quality of life before the treatment, we might measure the changes caused by the orthodontic treatment more accurately. To go further, prospective longitudinal studies could be conducted to explore the causal relationships. A second limitation is that the presence of orthodontic history was considered as a binary factor, and further studies could take the time after treatment into account, making the results more accurate. Besides, the PHQ-4 scale, although brief and easy to implement, might be a little bit sparse to indicate the real psychological status of the participant. Many detailed questionnaires could be used in the future to elaborately assess the subjects' psychological status.

## 5. Conclusion

This cross-sectional study showed around thirty percent of the included participants had orthodontic histories. Participants with OS had larger proportion of females, higher income, prevalence of TMD, and worse temporomandibular-related quality of life than those without OS. After adjusting possible confounding factors, OS was significantly associated with higher prevalence of TMD and worse temporomandibular-related quality of life, but not with the psychological status. The cause-and-effect relationship needs further exploration.

## Figures and Tables

**Table 1:** Demographic data of the participants.

	Without OS (*n* = 370)	With OS (*n* = 161)	*p* value
Age (year)	21.80 ± 2.77	22.00 ± 2.74	0.443

Sex			0.010
Male	166 (44.86%)	53 (32.92%)	
Female	204 (55.14%)	108 (67.08%)	

Education level			0.087
Undergraduate	233 (62.97%)	88 (54.66%)	
Master candidate	96 (25.95%)	45 (27.95%)	
Doctor candidate	41 (11.08%)	28 (17.39%)	

Family income per capita			<0.001
Less than 3000 yuan	107 (28.92%)	26 (16.15%)	
3000∼6000 yuan	149 (40.27%)	61 (37.89%)	
More than 6000 yuan	114 (30.81%)	74 (45.96%)	

Medical student			0.009
Yes	175 (47.30%)	96 (59.63%)	
No	195 (52.70%)	65 (40.37%)	

OS, orthodontic history.

**Table 2:** The psychological status of students with and without orthodontic history.

	Without OS	With OS	*p* value
Depression			0.390
No	311 (84.05%)	140 (86.96%)	
Yes	59 (15.95%)	21 (13.04%)	

Anxiety			0.504
No	312 (84.32%)	132 (81.99%)	
Yes	58 (15.68%)	29 (18.01%)	

PHQ total scores	2.90 ± 2.69	3.17 ± 2.28	0.276

**Table 3:** Binary regression models for the association between orthodontic history and presence of depression and anxiety.

	Nonadjusted	Adjust II
Depression		
Without OS	Reference	Reference
With OS	0.79 (0.46, 1.35), *p*=0.3909	0.98 (0.55, 1.74), *p*=0.9480

Anxiety		
Without OS	Reference	Reference
With OS	1.18 (0.72, 1.93), *p*=0.5040	1.16 (0.69, 1.96), *p*=0.5640

The nonadjusted model adjusts for none. The adjust II model adjusts for age, sex, income, education level, and major.

**Table 4:** The prevalence of TMD and the total scores of FAI and TMD-OHIP in the With OS and Without OS groups.

	Without OS	With OS	*p* value
TMD			0.003

No	219 (59.19%)	73 (45.34%)	

Yes	151 (40.81%)	88 (54.66%)	

Total scores of the FAI	17.70 ± 16.91	22.08 ± 18.09	0.007

Total scores of the TMD-OHIP	6.64 ± 10.79	9.64 ± 12.36	0.005

OHIP-1, functional limitation	0.77 ± 1.39	0.99 ± 1.31	0.093

OHIP-2, physical pain	1.41 ± 2.50	2.07 ± 2.88	0.008

OHIP-3, psychological discomfort	1.63 ± 2.59	2.39 ± 2.80	0.003

OHIP-4, physical disability	0.58 ± 1.11	0.96 ± 1.39	<0.001

OHIP-5, psychological disability	1.31 ± 2.56	1.91 ± 3.02	0.020

OHIP-6, social disability, mean	0.44 ± 0.98	0.63 ± 1.17	0.046

OHIP-7, handicap	0.51 ± 1.06	0.70 ± 1.27	0.075

**Table 5:** Binary regression models for the association between orthodontic history and presence of TMD.

	Nonadjusted	Adjust I	Adjust II
Without OS	Reference	Reference	Reference
With OS	1.75 (1.20, 2.54), *p*=0.0033	1.63 (1.11, 2.38), *p*=0.0121	1.67 (1.13, 2.47), *p*=0.0108

The nonadjusted model adjusts for none. The adjust I model adjusts for sex. The adjust II model adjusts for age, sex, income, education level, and major.

**Table 6:** Multiple linear regression models for the association between orthodontic history and scores of TMD-OHIP.

	Nonadjusted	Adjust I	Adjust II
Without OS	Reference	Reference	Reference
With OS	3.00 (0.91, 5.09), *p*=0.0051	3.46 (1.35, 5.57), *p*=0.0014	3.45 (1.30, 5.60), *p*=0.0018

The nonadjusted model adjusts for none. The adjust I model adjusts for income. The adjust II model adjusts for age, sex, income, education level, and major.

## Data Availability

The data used to support the findings of this study are available from the corresponding author upon reasonable request.

## Supplementary Materials

Supplementary Materials
*Supplementary Table 1*. The total scores of TMD-OHIP in participants with and without temporomandibular disorders.
