# Supplementary material for: Associations among Orthodontic History, Psychological Status, and Temporomandibular-Related Quality of Life: A Cross-Sectional Study
Source: Int J Clin Pract. 2022 May 28;2022:3840882. doi: 10.1155/2022/3840882 (PMC9167145; doi:10.1155/2022/3840882)
Supplement: Supplementary Materials — Supplementary Table 1. The total scores of TMD-OHIP in participants with and without temporomandibular disorders. [file 3840882.f1.docx]

**Supplementary table 1. The total scores of TMD-OHIP in participant with and with temporomandibular disorders.**

|  | No TMD (n= 292) | With TMD (n=239) | *p* value |
| --- | --- | --- | --- |
| Total scores of the TMD-OHIP | 2.96 ± 6.64 | 13.16 ± 13.27 | <0.001 |
| OHIP-1, Functional  Limitation | 0.30 ± 0.71 | 1.49 ± 1.67 | <0.001 |
| OHIP-2, Physical Pain | 0.57 ± 1.38 | 2.89 ± 3.18 | <0.001 |
| OHIP-3, Psychological  Discomfort | 0.76 ± 1.57 | 3.20 ± 3.10 | <0.001 |
| OHIP-4, Physical  Disability | 0.27 ± 0.79 | 1.21 ± 1.43 | <0.001 |
| OHIP-5, Psychological  Disability | 0.59 ± 1.78 | 2.59 ± 3.21 | <0.001 |
| OHIP-6, Social Disability,  Mean | 0.23 ± 0.78 | 0.82 ± 1.22 | <0.001 |
| OHIP-7, Handicap | 0.23 ± 0.78 | 0.97 ± 1.35 | <0.001 |
